# Depressive State in the Emergency Department During COVID-19: A National Cross-Sectional Survey in China

**DOI:** 10.3389/fpsyt.2021.566990

**Published:** 2021-06-14

**Authors:** Shuang Liu, Wei Han, Chenyu Shen, Changju Zhu, Qiaofang Wang, Xianquan Liang, Xiangxi He, Qin Xie, Jie Wei, Miao Wu, Xiaodong Zhao, Hongsheng Liu, Danping Liu, Xiaowang Guo, Shinan Nie, Liping Cao, Linxin Lu, Yaqin Fang, Zhongqiu Lu, Yixu Wu, Min Zhao, Jun Han, Xinchao Zhang, Jie Chang, Shuogui Xu, Wenjie Ma, Junli Si, Suxia Qi, Peng Peng, Yage Chai, Yu Cao, Yaowen Jiang, Wen Yin, Yanjun Wang, Hong Zhan, Yingxiong Huang, Ying Deng, Juanjuan Song, Lishan Yang, Jiali Wu, Banghan Ding, Danwen Zheng, Chuanyun Qian, Rui Huang, Jiyan Lin, Zhihong Xu, Guoxiu Zhang, Yingying Hu, Qingli Dou, Xiaoming Zhang, Yingping Tian, Dongqi Yao, Joseph Harold Walline, Huadong Zhu, Jun Xu, Yi Li, Xuezhong Yu

**Affiliations:** ^1^Department of Emergency Medicine, Peking Union Medical College Hospital, Chinese Academy of Medical Sciences, Beijing, China; ^2^Institute of Basic Medical Sciences, School of Basic Medicine, Peking Union Medical College, Chinese Academy of Medical Sciences, Beijing, China; ^3^Department of Psychiatric, Yuquan Hospital Tsinghua University, Beijing, China; ^4^Department of Emergency Medicine, First Affiliated Hospital of Zhengzhou University, Zhengzhou, China; ^5^Department of Emergency Medicine, The Second People's Hospital of Guiyang, Guiyang, China; ^6^Department of Intensive Care, Zhongnan Hospital of Wuhan University, Wuhan, China; ^7^Department of Emergency Medicine, Renmin Hospital of Wuhan University, Wuhan, China; ^8^Department of Emergency Medicine, Fourth Medical Center of PLA General Hospital, Beijing, China; ^9^Department of Emergency Medicine, Shaanxi Provincial People's Hospital, Xi'an, China; ^10^Department of Emergency Medicine, Jinling Hospital, Medical School of Nanjing University, Nanjing, China; ^11^Department of Emergency Medicine, Shanxi Bethune Hospital, Taiyuan, China; ^12^Department of Emergency Medicine, The First Affiliated Hospital of Wenzhou Medical University, Wenzhou, China; ^13^Department of Emergency Medicine, China Medical University of Shengjing Hospital, Shenyang, China; ^14^Department of Emergency Medicine, Beijing Hospital, Beijing, China; ^15^Department of Emergency Medicine, Changhai Hospital, Shanghai, China; ^16^Department of Emergency Medicine, Qingdao Municipal Hospital, Qingdao, China; ^17^Department of Emergency Medicine, The First Affiliate Hospital of Xinjiang Medical University, Wulumuqi, China; ^18^Department of Emergency Medicine, West China Hospital of Sichuan University, Chengdu, China; ^19^Department of Emergency Medicine, Xijing Hospital, Fourth Military Medical University, Xi'an, China; ^20^Department of Emergency Medicine, The First Affiliated Hospital, Sun Yat-sen University, Guangzhou, China; ^21^Department of Emergency Medicine, The Second Affiliated Hospital of Harbin Medical University, Harbin, China; ^22^Department of Emergency Medicine, General Hospital of Ningxia Medical University, Nanjing, China; ^23^Department of Emergency Medicine, Guangdong Provincial Hospital of Chinese Medicine, Guangzhou, China; ^24^Department of Emergency Medicine, The First Affiliated Hospital of Kunming Medical University, Kunming, China; ^25^Department of Emergency Medicine, The First Affiliated Hospital of Xiamen University, Xiamen, China; ^26^Department of Emergency Medicine, The First Affiliated Hospital and College of Clinical Medicine of Henan University of Science and Technology, Luoyang, China; ^27^Department of Emergency Medicine, People's Hospital of Shenzhen, Shenzhen, China; ^28^Department of Emergency Medicine, The Second Hospital of Hebei Medical University, Shijiazhuang, China; ^29^Accident and Emergency Medicine Academic Unit, Prince of Wales Hospital, The Chinese University of Hong Kong, Hong Kong, Hong Kong

**Keywords:** COVID-19, depression, emergency medicine, PHQ-9, China

## Abstract

Chinese emergency department (ED) staff encountered significant mental stress while fighting the coronavirus disease 2019 (COVID-19) pandemic. We sought to investigate the prevalence and associated factors for depressive symptoms among ED staff (including physicians, nurses, allied health, and auxiliary ED staff). A cross-sectional national survey of ED staff who were on duty and participated in combating the COVID-19 pandemic was conducted March 1–15, 2020. A total of 6,588 emergency medical personnel from 1,060 hospitals responded to this survey. A majority of respondents scored above 10 points on the PHQ-9 standardized test, which is associated with depressive symptoms. Those aged 31–45, those working in the COVID-19 isolation unit, and those with relatives ≤ 16 or ≥70 years old at home all had statistically significant associations with scoring >10 points. Depressive symptoms among Chinese emergency medical staff were likely quite common during the response to the COVID-19 pandemic and reinforce the importance of targeted ED staff support during future outbreaks.

## Introduction

At the end of December 2019, a new respiratory infection outbreak, later termed coronavirus disease 2019 (COVID-19), was first reported in Wuhan, China (1). Unfortunately, COVID-19 has continued to rampage throughout the world. According to the World Health Organization (WHO), there have already been hundreds of millions confirmed cases and several million deaths (2). The prevention and containment of COVID-19 have become issues of worldwide concern. Among a variety of control options, social distancing was recommended by the WHO to reduce the possibility of infection (3). Unfortunately, medical staff, particularly those on the frontlines of healthcare in emergency departments (EDs), have taken the brunt of the effort in the fight against COVID-19. They are unable to follow recommendations on social distancing and must work in areas that are high risk for COVID-19. According to data from the National Health Council of China, as of April 1, 2021, thousands of medical staff have been infected and many have died (4). ED staff are not only exposed to a higher risk of infection but also suffer the physical and mental strain of tiring work schedules, difficult triage decisions, fears of infecting family members, and the anguish of losing patients and colleagues to COVID-19 (5).

Previous studies showed that infectious disease pandemics, such as severe acute respiratory syndrome (SARS) and Middle East respiratory syndrome (MERS) can impact negatively on the mental health of different groups of people, including healthcare workers (6, 7). COVID-19 also likely results in psychological problems, such as stress, anxiety, and depressive symptoms, among frontline medical workers. Recently, several studies have reported that the prevalence of anxiety and depression among healthcare workers is higher during the COVID-19 pandemic (8, 9). However, research exploring the mental health problems of frontline medical workers in the ED is limited. The aim of this project was to examine the prevalence of depressive symptoms among ED medical personnel in China during the early (and most severe phase so far for China) of the COVID-19 pandemic.

## Materials and Methods

### Study Design and Participants

This was a national cross-sectional survey conducted between March 1 and March 15, 2020. The study was approved by the Ethics Committee of Peking Union Medical College Hospital, and all participants provided informed consent. Chinese ED staff (including physicians, nurses, allied health, and auxiliary ED staff) between 18 and 80 years of age who were on clinical duty in areas designated to receive COVID-19 patients between November 1, 2019, and March 15, 2020, were invited to participate. Only those able to complete informed consent were eligible for inclusion. Anyone previously diagnosed with any mental illness, those taking any antipsychotic medications, or those participating in other clinical trials were excluded. Due to the sudden outbreak of COVID-19 in China, some retired medical staff participated in the fight against the pandemic, so our study included medical staff over 60 years old (the normal retirement age in China). Finally, since nearly all ED staff on clinical duty in China during this period participated in fighting against the COVID-19 pandemic work, a concurrent control group of ED staff who did not participate in the fight against the pandemic was not feasible.

### Survey Instrument

Our survey instrument begins with collecting respondents' general characteristics, including sex, age, profession, relationship status, and whether they live with children younger than 16 years old or adults older than 70 years old.

We then queried respondents' work details during COVID-19. Specifically, we asked whether they worked in Hubei province (where the city of Wuhan is located, the site of the most significant COVID-19 outbreak in mainland China during the study period). During the pandemic, many medical staff across the country left their long-term work locations and went to Hubei to participate in fighting against the COVID-19 pandemic work, so we divided people working in Hubei into two groups: those who were living in Hubei for at least 2 years before the outbreak and those who went to Hubei to participate in anti-pandemic work. We also queried whether respondents worked in COVID-19 isolation wards, whether they were directly in contact with any confirmed COVID-19 patients (regardless of any protective measures being taken), whether they underwent compulsory isolation in their hospital due to workplace exposure to COVID-19 (i.e., they were being quarantined in the hospital facilities not due to being sick themselves), and the duration of such isolation time. We additionally clarified this issue in our research, by dividing the medical staff into “compulsory isolation in the hospital (i.e., they still needed to stay in the hospital when they are not at work)” and “non-compulsory isolation in the hospital (they could go home after work).”

We then asked respondents about the time spent working during the COVID-19 outbreak: what their working hours were before and after the outbreak of COVID-19 (including the average working hours of each rotation and weekly working hours). We collected the working hours of medical staff between November 2019 to March 2020 (the official “winter” months in China) and selected January 15, 2020, as date of the outbreak of China's COVID-19 pandemic. January 15, 2020, was the infection point for COVID-19 cases in Wuhan (the average number of daily hospital admissions for fever jumped from 300 to 600 that day).

### Patient Health Questionnaire-9 (PHQ-9)

After the collection of respondent characteristics and work details, the Patient Health Questionnaire-9 (PHQ-9) standardized questionnaire was used to ascertain the psychological state of surveyed ED staff. The Patient Health Questionnaire depression module is a self-rated version of the Primary Care Evaluation of Mental Disorders Patient Questionnaire (PRIME-MD PQ) for depression (10, 11). PHQ-9 has been validated in two large studies involving 3,000 patients in seven obstetrics and gynecology clinics and another study with 3,000 patients in eight primary care clinics (12). This scale scores each of the nine diagnostic criteria for depression in the DSM-IV on a scale from “0” (not at all) to “3” (nearly every day) (12). The PHQ-9 is scored 0–27, with the interpretation based on the following intervals: 0–4, 5–9, 10–14, 15–19, and 20–27. The cutoff score for major depression symptoms in prior studies was set at 10, with subjects scoring higher than 10 being defined as having depressive symptoms. Using the Mental Health Professional (MHP) Validation Interviews as the criterion standard, a PHQ-9 score ≥10 had a sensitivity of 88% and a specificity of 88% for major depression (13), while a PHQ-9 score <10 yielded a negative predictive value of 0.99 (14). PHQ-9 has been widely applied in clinical institutions and scientific research to assist in making the diagnosis of depression, quantify depressive symptoms, and monitor their severity. We utilized the standard score of ≥10 as the critical value to divide those with or without a depressed state in this study. We hypothesized that most respondents would have a score >10.

### Survey Process

In this study, we combined PHQ-9 with our own queries for respondent characteristics and work details as noted above. We then used an online questionnaire system (Gold Data, Jingshuo Technology Corporation, Beijing, China) as the platform for distributing our survey tool. We pushed out the survey instrument in an online “snowball” method of sampling by sending the survey out through friendship circles and promotion through emergency medicine groups on the WeChat messaging platform (Tencent Corporation, Shenzhen, China). The Gold Data system was then able to collect the survey data electronically.

### Statistical Analysis

Continuous variables were described with mean and standard deviation, while categorical variables were described by frequency and percentages. When the distribution of a continuous variable was skewed, the median and interquartile ranges were presented. Student's *T*-tests or one-way analysis of variance was employed for two groups or multiple-group continuous-measure comparisons, as appropriate. Chi-square tests were used for comparing categorical measures. Multiple logistic regression models were used to estimate the odds ratio (OR) and 95% confidence interval (CI) for the association between associated factors and depressive symptoms, with the risk factors selected by a forward stepwise method. All analyses were conducted using SAS version 9.2 (SAS Institute, Inc., Cary, NC, USA). A *p* < 0.05 was considered statistically significant.

## Results

We received 7,000 completed questionnaires through the online survey system, of which 6,588 (94.00%) were valid. Respondents came from 1,060 hospital EDs in 27 (out of a surveyed 31) provinces, autonomous regions, or independent municipalities in China. The average PHQ-9 score for all medical staff was 10.94 ± 5.1, and 3,795 out of 6,588 participants (57.60%) had a PHQ-9 score ≥10. The prevalence of depressive symptoms was high with a PHQ-9 score distribution of 10–14 (34.44%), 15–19 (16.27%), and 20–27 (6.89%).

### Participant Characteristics

Participant characteristics are shown in Tables 1, 2. Among these samples, 33.58% were male and 66.42% were female. Most respondents (87.56%) were ≤ 45 years old. Among the medical staff surveyed, nurses and doctors accounted for 59.01 and 38.29%, respectively. Almost all (95.1%) participants were from outside of Hubei province during the COVID-19 outbreak. In addition, 56.70% of participants had children ≤ 16 years old at home who needed care, while 31.21% of participants lived with elderly family members ≥70 years old.

**Table 1:** Participant characteristics.

**Characteristic**	**Number (percent)**
	**(*N* = 6,588)**
**Gender**	
Male	2,212 (33.58%)
Female	4,370 (66.42%)
**Age (y)**	
18–30	2,713 (41.18%)
31–45	3,056 (46.38%)
46–60	798 (12.11%)
>60	21 (0.31%)
**Occupation**	
Medical	2,523 (38.29%)
Nurse	3,888 (59.01%)
Allied	150 (2.27%)
Auxiliary	27 (0.40%)
**Marital status**	
Married	4,665 (70.81%)
Single	1,770 (26.86%)
Divorced or widowed	153 (2.32%)
**Living with children** **≤16 y of age**	
Yes	3,737 (56.72%)
No	2,849 (43.28%)
**Living with adult** **≥70 y of age**	
Yes	2,056 (31.20%)
No	4,532 (68.80%)
**Working area in the COVID-19 period**	
Hubei province	325 (4.90%)
Other provinces	6,263 (95.10%)
**Working status in the COVID-19 period**	
Long-term work in Hubei	84 (1.20%)
Supporting Hubei work	241 (3.65%)
Work in other provinces	6,263 (95.15%)
**Working in the isolation unit with COVID-19 patients**	
Yes	3,021 (45.85%)
No	3,565 (54.11%)
**Direct contact with confirmed COVID-19 patients**	
Yes	1,029 (15.61%)
No	5,559 (84.38%)
**Compulsory isolation in hospital**	
Yes	6,122 (92.92%)
No	466 (7.08%)
**Distribution of PHQ-9 scores**	
0–4	547 (8.30%)
5–9	2,246 (34.09%)
10–14	2,269 (34.44%)
15–19	1,072 (16.27%)
20–27	454 (6.89%)

**Table 2:** Univariate analysis results between the PHQ-9 < 10 and PHQ-9 ≥ 10 groups.

**Characteristic**	**Overall**	**Overall PHQ score**	**PHQ-9 <10**	**PHQ-9 ≥ 10**	*P*-value^*^
	**(*N* = 6,588)**		**(*N* = 2,793)**	**(*N* = 3,795)**	
		**Mean ± SD**	***N* (%)**	***N* (%)**	
**Gender**					
Male	2,212	11.2 ± 5.3	899 (40.64%)	1,313 (59.36%)	0.0396
Female	4,370	10.8 ± 5.0	1,892 (43.30%)	2,478(56.70%)	
**Age (y)**					
18–30	2,713	10.3 ± 4.9	1,268 (46.74%)	1,445 (53.26%)	
31–45	3,056	11.3 ± 5.2	1,200 (39.27%)	1,856 (60.73%)	<0.0001
46–60	798	11.4 ± 5.3	312 (39.10%)	486 (60.90%)	
>60	21	9.7 ± 7.0	13 (61.90%)	8 (38.10%)	
**Occupation**					
Medical	2,523	11.4 ± 5.3	981 (38.88%)	1,542 (61.12%)	<0.0001
Nurse	3,888	10.7 ± 5.0	1,714 (44.08%)	2,174 (55.92%)	
Allied	150	9.5 ± 5.3	82 (54.67%)	68 (45.33%)	
Auxiliary	27	9.2 ± 5.0	16 (59.26%)	11 (40.74%)	
**Marital status**					
Married	4,665	11.1 ± 5.1	1,899 (40.71%)	2,766 (59.29%)	<0.0001
Single	1,770	10.5 ± 4.9	830 (46.89%)	940 (53.12%)	
Divorced or widowed	153	11.5 ± 5.9	64 (41.83%)	89 (58.17%)	
**Living with children** **≤16 y of age**					
Yes	3,737	11.3 ± 5.2	1,475(39.47%)	2,262 (60.53%)	<0.0001
No	2,849	10.5 ± 4.9	1,317 (46.23%)	1,532 (53.77%)	
**Living with adult(s)** **≥70 y of age**					
Yes	2,056	10.6 ± 4.9	740 (35.99%)	1,316 (64.01%)	<0.0001
No	4,532	11.8 ± 5.3	2,053 (45.30%)	2,479 (54.70%)	
**Working area**					
Hubei province	325	10.2 ± 5.1	153 (47.08%)	172 (53.92%)	0.0798
Other provinces	6,263	11.0 ± 5.1	2,640 (42.15%)	3,623 (57.85%)	
**Working status**					
Long-term work in Hubei	84	11.1 ± 5.4	35 (41.67%)	49 (58.33%)	0.1094
Supporting Hubei work	241	9.9 ± 4.9	118 (48.96%)	123 (51.04%)	
Work in other provinces	6,263	11.0 ± 5.1	2,640 (42.15%)	3,623 (57.85%)	
**Working in the isolation unit with COVID-19 patients**					
Yes	3,021	11.6 ± 5.3	1,134 (37.54%)	1,887 (62.46%)	<0.0001
No	3,565	10.3 ± 4.8	1,658 (46.51%)	1,907 (53.49%)	
**Direct contact with confirmed COVID-19 patients**					
Yes	1,029	11.6 ± 5.2	385 (37.41%)	644 (62.59%)	0.0004
No	5,559	10.8 ± 5.1	2,408 (43.32%)	3,151 (56.68%)	
**Compulsory isolation in hospital**					
Yes	6,122	11.4 ± 4.9	2,621 (42.81%)	3,501 (57.19%)	0.0129
No	466	10.9 ± 5.1	172 (36.91%)	294 (63.09%)	

* *χ^2^test*.

Almost half (45.86%) of the ED staff who participated in this study worked in their hospital's COVID-19 isolation area(s), and 15.62% of them had direct contact with patients known to be infected with COVID-19. Almost all (92.93%) ED staff were forced to quarantine in their hospital while on service.

### Factors Associated With Major Depressive Symptoms Among ED Staff

Results of univariate analysis are shown in Table 2. Severe depressive symptoms divided by prevalence according to age group, occupation, and marital status were all statistically significant. In addition, the prevalence of PHQ-9 scores ≥10 was higher in males compared to females (*p* = 0.040). Similar results were found in participants who lived with children ≤ 16 and adults ≥70, those who worked in the COVID-19 isolation unit, and those who had direct contact with COVID-19 patients (*p* < 0.05). There was no significant difference in the PHQ-9 scores between people who were or were not working in Hubei province during the outbreak.

### Duty Time and Quarantine of the ED Staff

Among all respondents, the average time per duty rotation was 10 h, and the average time at work per week was nearly 50 h. There was no difference in the number of hours worked in Hubei compared to other provinces (working hours per week, 50.38 ± 22.0 vs. 48.34 ± 18.6, *p* = 0.101; working hours per rotation, 11.78 ± 8.0 vs. 11.89 ± 7.6, *p* = 0.799). There was no difference in the average daily work hours per shift before or during the pandemic among respondents (11.88 ± 7.4 vs. 11.89 ± 7.6, *p* = 0.850). However, the average weekly work hours before COVID-19 were more than the average hours during COVID-19 (49.49 ± 18.5 vs. 48.44 ± 18.8, *p* < 0.001).

In our survey, the ED staff who were forced to quarantine in their hospital had a higher PHQ-9 score than those who were not forced into hospital isolation (11.4 ± 4.9 vs. 10.9 ± 5.1, *p* = 0.0129; Table 2). In addition, there was no statistically significant difference in scores between groups that had more or <14 quarantine days (PHQ score, 11.63 ± 5.1 vs. 11.35.9 ± 4.8, *p* = 0.970).

### Factors Associated With Depressive Symptoms by Multivariate Analysis

As shown previously, univariate analysis (Table 2) revealed several variables associated with a PHQ-9 score ≥10. Subsequent multiple logistic regression analysis showed that a score ≥10 during the COVID-19 pandemic was significantly associated with direct contact with confirmed COVID-19 patients (OR = 1.153, 95% CI: 0.994–1.338), working in the COVID-19 isolation unit (OR = 1.366, 95% CI: 1.23–1.517), respondents between 31 and 45 years of age (OR = 1.139, 95% CI: 1.004–1.293), and those staff living with children ≤ 16 years old (OR = 1.126, 95% CI: 1.001–1.267) or adults ≥70 years old (OR = 1.325, 95% CI: 1.177–1.492). Results of the multivariate logistic regression analysis are shown in Table 3.

**Table 3:** Factors associated with depressive symptoms by multivariate analysis.

	**Coefficients**	SE^*^	Statistics^**^	***P*-value**	**OR^**†**^**	**95% CI^**‡**^**
Direct contact with confirmed COVID-19 patients	0.142	0.0758	3.5206	0.0606	1.153	0.994–1.338
Working in the isolation unit with COVID-19 patients	0.312	0.0534	34.1538	<0.0001	1.366	1.23–1.517
Age between 18 and 30 years old	Ref					
Age between 31 and 45 years old	0.130	0.0645	4.0756	0.0435	1.139	1.004–1.293
Age between 46 and 60 years old	0.054	0.0935	0.333	0.5639	1.055	0.879–1.268
Age above 60 years old	−0.834	0.4593	3.2987	0.0693	0.434	0.176–1.068
living with children ≤ 16 y of age	0.1189	0.0599	3.936	0.0473	1.126	1.001–1.267
Living with adult ≥70 y of age	0.2818	0.0604	21.7272	<0.0001	1.325	1.177–1.492

* *Standard error of estimated coefficients*.

** *χ^2^statistics*.

† *Odds ratio*.

‡ *95% confidence interval for odds ratio*.

## Discussion

This was a large-scale, multicenter, cross-sectional study of the prevalence and risk factors for depression among medical staff during the COVID-19 pandemic. Because the population surveyed in this study covered most provinces and cities in China, we can draw a relatively complete picture of the prevalence of depressive symptoms among Chinese ED staff during the fight against the COVID-19 pandemic. The results of this study show that more than half of all staff surveyed experienced PHQ-9 scores ≥10, and such elevated scores were associated with age, family factors, and exposure to COVID-19 patients and were independent of work time or location. These results offer a comprehensive national assessment of potential depressive state in ED staff that may be used to guide future mental health improvement efforts.

Many recent psychological investigations on health professionals during COVID-19 have shown that health professionals fighting COVID-19 are suffering from more psychiatric disorders than other occupational groups (8, 15, 16). Wang et al. (17) performed a survey of Chinese physicians in Liaoning province and found that the prevalence of depressive symptoms among doctors was 65.3%. Lai et al. (8) performed a multicenter cross-sectional survey which collected demographic data and mental health measures of 1,257 health professionals that treat patients exposed to COVID-19 in China, and they reported that 50.4% showed symptoms of depression. The rate of depressive state (PHQ-9 score ≥10) was 57.60% in our study. The proportion of people with depressive symptoms is slightly higher than the results of previous studies. One possible reason for this is that our study included only ED staff, whereas participants in other studies were from a variety of other specialties (e.g., respiratory medicine, critical care medicine, other internal medicine specialties, or anesthesia). In China, medical staff in the ED have often already experienced high levels of stress even before COVID-19 began due to heavy workloads under uncertain conditions.

In most previous studies on the psychological impact of the COVID-19 pandemic on frontline healthcare workers, female nurses with close contact with COVID-19 patients appeared to have the highest mental health risks (18–21). It is important to note that most previous studies included predominantly female participants, particularly nurses. Like the population composition of most previous studies, female participants also accounted for most of the population in our survey, but male respondents on average had higher PHQ-9 scores than female respondents. While the reasons behind this result are still unclear, this does help illustrate that female respondents do not have a monopoly on depression. Among other factors related to depressive symptoms among the ED staff surveyed, our correlation analysis indicated that a younger age and a married marital status were associated with depression. These predictors were mostly consistent with previous research (22, 23). The 31–45-year-old age group accounted for the largest proportion of subjects as well as the subgroup with the highest PHQ-9 scores. Compared with younger doctors, senior medical staff with more work experience may have more experience dealing with complex situations, which could explain their lower perceived stress and better resilience (24).

Isolation for ED staff during the COVID-19 outbreak was an additional stressor for frontline medical staff. During the COVID-19 period, the Chinese government strongly recommended that everyone reduce travel and self-quarantine as much as possible in their current place of residence, so it can be difficult to clearly define any additional “compulsory” element of isolation. In this study, we further divided any isolating ED staff into those who reported “compulsory isolation” in their hospital (they needed to remain on hospital property even when they are not on duty) and “non-compulsory isolation” outside of their hospital (they could go home after work). Isolation and confinement during the epidemic could cause a loss of daily habits, reduce socialization with other people, and directly lead to boredom and depression (25). ED staff who were not isolated in the hospital could still live at home, communicate with family and friends, and continue to receive their family's emotional support and encouragement. In addition, ED staff who were forced to be isolated at their hospital may be in close contact with infected patients, thereby aggravating social stigma. In our study, the average PHQ-9 score and the proportion of PHQ-9 scores ≥10 were indeed higher in those ED staff who had to isolate in the hospital compared to those who could go home after work.

Surprisingly, compared with those inside Hubei province, those outside of Hubei province had no significant difference in PHQ-9 scores, this is different from the results of other cross-sectional studies during the same period, which showed that medical staff deployed to Hubei province had a higher prevalence of depressive symptoms than physicians and nurses working in fever clinics and infectious disease wards outside of Hubei province (8, 22, 26–28). During the outbreak of COVID-19, physicians and nurses deployed to Hubei province had to face confirmed COVID-19 patients. They had to work in unfamiliar environments, the patients they saw every day were more critical, and many of them needed immediate care (26, 27). In our study, ED medical personnel working in Hubei made up a relatively small portion (4.93%) of the overall study population, and this may prevent a meaningful analysis of their risk profile, being a limitation to the present study. Working in Wuhan was associated with more stress, but statistical significance was not met, possibly due to an insufficient sample size.

Looking at duty hours, we found that, regardless of the pandemic, the average working hours of all Chinese ED staff are relatively long. The average shift is 12 h long, and the average work hours per week are close to 50 h. High-intensity and time-consuming work may cause medical staff to become fatigued, resulting in higher overall PHQ-9 scores.

During the COVID-19 outbreak, there was no difference in both the number of duty hours per day and the number of duty hours per week in different regions (including Hubei province). This indicates that even though Hubei was the epicenter in China's fight against the pandemic and there were more COVID-19 patients there than in other provinces, there seems to have been no serious imbalance in work hours compared to elsewhere in China. In addition, the duty hours for staff in other provinces did not decrease either. The number of medical staff in other provinces may have declined due to transfers to Hubei, but, due to fewer ED cases throughout the country, the remaining workers seemed to be on duty about the same amount of time. The workload of first-line medical staff in provinces other than Hubei should therefore not be minimized.

In a related point, the total number of work hours per week for ED staff was less after the COVID-19 outbreak than before. There are many reasons for this, including additional medical staffing support from other departments, thereby reducing the average work hours for emergency medicine staff. National policy required medical staff who worked in the COVID-19 isolation ward(s) to have 14 days of compulsory isolation, which may also have reduced the average number of hours worked. During the COVID-19 period, the number of patients with fever increased significantly compared to previous years, and the number of patients who went to the hospital for other diseases was much smaller than usual. Similar situations have been reported in other regions. For example, from February 1 to April 30, 2020, the number of ED patients in Hong Kong decreased by 37% (28).

Our study had several limitations. First, like other screening questionnaires, the PHQ-9 scale is not sufficiently accurate to establish a definitive diagnosis of major depression. Scores exceeding the threshold are, in effect, a positive screen which should prompt a careful mental health assessment. Even though a score ≥10 does not equal major depression, high score may lead to other diagnoses that share symptoms with major depression, such as anxiety disorder, alcohol use disorder, or subsyndromal depression. Second, although we have obtained correlations for many single-factor analyses, many variables related to depression have not yet been explored. The correlation between different variables requires the creation of a comprehensive variable, which can be directly linked to the PHQ-9 score. Finally, this was a cross-sectional study and cannot directly establish the relationship between depression and related factors.

In conclusion, our study showed that most Chinese ED staff who worked clinically during the response to COVID-19 had elevated PHQ-9 scores which put them at a very high risk for major depression. This is the first comprehensive study to explore the prevalence and associated factors of depression among emergency medicine workers in Chinese EDs. Policymakers should implement appropriate proactive interventions for ED staff in times of extreme distress, either during COVID-19 outbreaks or during future pandemics.

## Data Availability Statement

The raw data supporting the conclusions of this article will be made available by the authors, without undue reservation.

## Ethics Statement

The studies involving human participants were reviewed and approved by Ethics Committee of Peking Union Medical College Hospital. The patients/participants provided their written informed consent to participate in this study.

## Author Contributions

SL and XY contributed to the study design. SL, CZ, XL, QX, JW, XDZ, DL, SN, LL, ZL, MZ, XCZ, QGX, JLS, PP, YC, WY, HZ, YD, LY, BD, CQ, JL, GZ, QD, and YT contributed to the data collection. QW, XH, MW, HL, XG, LC, YF, YXW, JH, JC, WM, SQ, YGC, YJ, YJW, YXH, JJS, JLW, DZ, RH, ZX, YYH, and DY contributed to the data entry. HDZ, JX, YL, and XY contributed to study monitoring. SL, WH, and CS contributed to the data interpretation and analysis. SL and CS contributed to the literature search. SL, CS, XMZ, and JHW contributed to the writing. All the authors read and approved the final version of the manuscript.

## Conflict of Interest

The authors declare that the research was conducted in the absence of any commercial or financial relationships that could be construed as a potential conflict of interest.

## References

[1] Zhu N Zhang D Wang W Li X Yang B Song J. A novel coronavirus from patients with pneumonia in China, 2019. N Engl J Med. (2020) 382:727–33. 10.1056/NEJMoa2001017 31978945 PMC7092803

[2] Update on Coronavirus 2019 (COVID-19) situation dashboard. Available online at: https://who.sprinklr.com/

[3] Coronavirus disease 2019 (COVID-19) advice for the public. Available online at: https://www.who.int/emergencies/diseases/novel-coronavirus-2019/advice-for-public

[4] Official website of the National Health Committee of the people's republic of China. Available online at: http://www.nhc.gov.cn/

[5] The Lancet. COVID-19: protecting health-care workers. Lancet. (2020) 395:922. 10.1016/S0140-6736(20)30644-9 PMC7138074 32199474

[6] Jeong H Yim HW Song YJ Ki M Min JA Cho J. Mental health status of people isolated due to Middle East respiratory syndrome. Epidemiol Health. (2016) 38:e2016048. 10.4178/epih.e2016048 28196409 PMC5177805

[7] Lu YC Shu BC Chang YY Lung FW. The mental health of hospital workers dealing with severe acute respiratory syndrome. Psychother Psychosom. (2006) 75:370–5. 10.1159/000095443 17053338

[8] Lai J Ma S Wang Y Cai ZX Hu JB Wei N. Factors associated with mental health outcomes among health care workers exposed to coronavirus disease 2019. JAMA Netw Open. (2020) 3:e203976. 10.1001/jamanetworkopen.2020.3976 32202646 PMC7090843

[9] Lu W Wang H Lin Y Li L. Psychological status of medical workforce during the COVID-19 pandemic: a cross-sectional study. Psychiatry Res. (2020) 288:112936. 10.1016/j.psychres.2020.112936 32276196 PMC7195354

[10] Löwe B Kroenke K Herzog W Kerstin G. Measuring depression outcome with a brief self-report instrument: sensitivity to change of the Patient Health Questionnaire (PHQ-9). J Affect Disord. (2004) 81:61–6. 10.1016/S0165-0327(03)00198-8 15183601

[11] Tamburrino MB Lynch DJ Nagel RW Smith MK. Primary care evaluation of mental disorders (PRIME-MD) screening for minor depressive disorder in primary care. Primary Care Compan J Clin Psychiatry. (2009) 11:339–43. 10.4088/PCC.08.m00711 20098526 PMC2805570

[12] Spitzer RL Williams JBW Kroenke K Hornyak R McMurry J. Validity and utility of the Patient Health Questionnaire in assessment of 3000 obstetric-gynecologic patients: the PRIME-MD Patient Health Questionnaire Obstetrics-Gynecology study. Am J Obstet Gynecol. (2000) 183:759–69. 10.1067/mob.2000.106580 10992206

[13] Spitzer RL Kroenke K Williams JBW. Patient Health Questionnaire study group. Validity and utility of a self-report version of PRIME-MD: the PHQ primary care study. JAMA. (1999) 282:1737–44. 10.1001/jama.282.18.1737 10568646

[14] Kroenke K Spitzer RL Williams JB. The PHQ-9: validity of a brief depression severity measure. J Gen Intern Med. (2001) 16:606–13. 10.1046/j.1525-1497.2001.016009606.x 11556941 PMC1495268

[15] Zhang WR Wang K Yin L Zhao WF Xue Q Peng M. Mental health and psychosocial problems of medical health workers during the COVID-19 epidemic in China. Psychother Psychosom. (2020) 89:242–50. 10.1159/000507639 32272480 PMC7206349

[16] da Silva FCT Neto MLR. Psychiatric symptomatology associated with depression, anxiety, distress, and insomnia in health professionals working in patients affected by COVID-19: a systematic review with meta-analysis. Prog Neuropsychopharmacol Biol Psychiatry. (2021) 104:110057. 10.1016/j.pnpbp.2020.110057 32777327 PMC7411383

[17] Wang JN Sun W Chi TS Wu H Wang L. Prevalence and associated factors of depressive symptoms among Chinese doctors: a cross-sectional survey. Int Arch Occup Environ Health. (2010) 83:905–11. 10.1007/s00420-010-0508-4 20112108

[18] Pouralizadeh M Bostani Z Maroufizadeh S Ghanbari A Khoshbakht M Alavi SA. Anxiety and depression and the related factors in nurses of Guilan University of Medical Sciences hospitals during COVID-19: a web-based cross-sectional study. Int J Afr Nurs Sci. (2020) 13:100233. 10.1016/j.ijans.2020.100233 32837911 PMC7417274

[19] Romero CS Delgado C Catalá J Ferrer C Errando C Iftimi A. PSIMCOV group^*^. COVID-19 psychological impact in 3109 healthcare workers in Spain: the PSIMCOV group. Psychol Med. (2020) 14:1–7. 10.1017/S0033291720001671 32404217 PMC7477466

[20] Elbay RY Kurtulmuş A Arpacioglu S Karadere E. Depression, anxiety, stress levels of physicians and associated factors in Covid-19 pandemics. Psychiatry Res. (2020) 290:113130. 10.1016/j.psychres.2020.113130 32497969 PMC7255248

[21] Cabarkapa S Nadjidai SE Murgier J Ng CH. The psychological impact of COVID-19 and other viral epidemics on frontline healthcare workers and ways to address it: a rapid systematic review. Brain Behav Immun Health. (2020) 8:100144. 10.1016/j.bbih.2020.100144 32959031 PMC7494453

[22] Wang LQ Zhang M Liu GM Nan SY Li T Xu L. Psychological impact of coronavirus disease (2019) (COVID-19) epidemic on medical staff in different posts in China: a multicenter study. J Psychiatr Res. (2020) 129:198–205. 10.1016/j.jpsychires.2020.07.008 32763586 PMC7834267

[23] Sareen J Erickson J Medved MI Asmundson GJ Enns MW Stein M. Risk factors for post-injury mental health problems. Depress Anxiety. (2013) 30:321–7. 10.1002/da.22077 23408506

[24] BeyondBlue. National Mental Health Survey of Doctors and Medical Students. Melbourne, QLD: BeyondBlue; The National Depression Initiative (2013).

[25] Brooks SK Webster RK Smith LE Woodland L Wessely S Greenberg N. The psychological impact of quarantine and how to reduce it: rapid review of the evidence. Lancet. (2020) 395:912–20. 10.1016/S0140-6736(20)30460-8 32112714 PMC7158942

[26] Chen N Zhou M Dong X Qu J Gong F Han Y. Epidemiological and clinical characteristics of 99 cases of 2019 novel coronavirus pneumonia in Wuhan, China: a descriptive study. Lancet. (2020) 395:507–13. 10.1016/S0140-6736(20)30211-7 32007143 PMC7135076

[27] Guan WJ Ni ZY Hu Y Liang WH Ou CQ He JX China Medical treatment expert group for Covid-19. Clinical characteristics of coronavirus disease 2019 in China. N Engl J Med. (2020) 382:1708–20. 10.1056/NEJMc2005203 32109013 PMC7092819

[28] Walline JH Song PP Lim AM Hung KK Graham CA. Hong Kong emergency department attendance plummets during COVID-19. Emerg Med Australas. (2020) 32:1093–4. 10.1111/1742-6723.13614 32864889

